# Acute flaccid paralysis incidence rate and epidemiology in children in Lebanon: a rise in numbers in the post-vaccination and refugee crisis era

**DOI:** 10.4314/ahs.v22i2.14

**Published:** 2022-06

**Authors:** Said El Hage, Steven Safi, Elise Assouad, Antonio El Kareh, Elie Mokled, Pascale Salameh

**Affiliations:** 1 Faculty of Medical Sciences, Lebanese University, Hadath, Lebanon; 2 Neuroscience Research Center, Faculty of Medical Sciences, Lebanese University, Hadath, Lebanon; 3 School of Medicine, Lebanese American University, Byblos, Lebanon; 4 INSPECT-LB (Institut Nationale de Santé Publique, Epidémiologie Clinique et Toxicologie – Liban), Beirut, Lebanon; 5 University of Nicosia Medical School, Nicosia, Cyprus

**Keywords:** Acute flaccid paralysis, Epidemiology, Guillain-Barré Syndrome, Lebanon, Pediatrics

## Abstract

**Background:**

Acute flaccid paralysis (AFP) is a clinical syndrome characterized by the acute onset of weakness and paralysis with reduced muscle tone. This study explored the incidence and different aspects of AFP in Lebanese children between 2009 and 2019.

**Methods:**

AFP data were collected from the Lebanese Ministry of Public Health. Incidence rate according to year, age groups, clinical data, follow-up, diagnosis, and vaccination status was analyzed in the 11-years period.

**Results:**

AFP incidence rates increased importantly from 0.63 per 100,000 in 2009 till 4.96 per 100,000 in 2019. Most of the patients were children under ten years of age, 40.6% of all cases were under five years old, and 37.9% were between 5 and 9 years old. Follow-up revealed that approximately two out of five patients experienced residual weakness. As for the final diagnosis, around 30% of cases were diagnosed as Guillain-Barre Syndrome (GBS). Most cases were children having received between 3 and 5 doses of polio vaccine.

**Conclusions:**

The rise in cases coincided with the Syrian refugee crisis in Lebanon and the progressively deteriorating economy in the country; yet, incidence rates were in the lower margin compared with other countries.

## Introduction

Acute flaccid paralysis (AFP) is a rare clinical syndrome, having various infectious and non-infectious causes, with a broad array of potential etiologies that vary with age. AFP is characterized by the acute onset of weakness and paralysis with reduced muscle tone^1^. In the pre-vaccine era, poliomyelitis was the leading cause of AFP^2^. However, multiple medical conditions can also evolve into AFP, such as lesions of the anterior horn cells, Guillain-Barré syndrome, diphtheria, myositis, and infections by enterovirus D68. Besides, other central nervous system pathologies can cause AFP, such as spinal infarcts, neuromyelitis Optica (NMO), spinal arteriovenous fistulas, spinal epidural hematomas, spinal parasitic infections, and others^3^,^4^. According to the WHO, AFP is characterized by a sudden onset of paralysis/weakness in any part of the body, often involving the muscles that are responsible for respiration and swallowing, which progresses to maximum severity within 1 to 10 days^5^,^6^. Although AFP may affect individuals of any age, it predominantly affects children younger than 15 years old. AFP cases can be classified as polio-related AFP or non-polio AFP, according to laboratory exams of the patient or any close contact, by isolating a wild poliovirus (AFP surveillance guidelines, MOPH).

In spite of its rarity and despite the Global Polio Eradication Initiative declared by WHO in the 20^th^ century^7^, acute flaccid paralysis due to vaccine-associated poliomyelitis is still evident in Papua New Guinea in 2018^8^. Large outbreaks of acute flaccid myelitis due to non-polio enteroviruses (often enterovirus A71) have occurred, particularly in South East Asia^9^.

In the Middle-Eastern region, polio outbreaks have also erupted, often leading to AFP. In 2017, Syria was the victim of a vaccine-derived poliovirus type 2, resulting in 74 cases of AFP, the most recent of which showing onset of paralysis on 21 September 2017^10^.

On the local scale, the Lebanese MOPH's ESU (https://www.moph.gov.lb) has established an AFP surveillance program that reports cases of AFP since 2009. The registry categorizes reported cases according to many factors, including age group, region, and clinical data. In recent years, the reported AFP cases have reached relatively high numbers, and as far as we know, no previous studies have investigated the different aspects of AFP cases.

## Aim

We aim to calculate AFP incidence rates in Lebanon between 2009 and 2019 and assess AFP's distribution according to sex, age, and region. We will also compare the incidence rates in Lebanon to the rates of other countries, categorized as regional countries and randomly selected countries.

## Methods

### Study design and database details

This study is a retrospective descriptive epidemiological study performed using tables provided by the Lebanese Ministry of Public Health's Epidemiological Surveillance Unit (MOPH's ESU) from the AFP surveillance system and publicly available on the MOPH official website. The database was screened between 2009–2019, and data were specifically selected to match the study's aim.

### Case definition

Reported cases of AFP are classified as suspected, possible, and confirmed polio or non-polio cases according to laboratory tests and criteria listed in the MOPH's AFP Surveillance Guideline^12^, a document publicly available on the MOPH's official website. Laboratory tests include stool specimen examination.

Children under 15 years of age presenting AFP for any other reason than severe trauma are suspected cases of poliomyelitis. Paralytic illness in a person of any age, in whom the physician suspects polio can also be considered a suspected case.

Cases are classified according to laboratory results as polio-confirmed, polio-compatible, or polio-discarded cases.

### Polio-confirmed cases

The laboratory isolates a wild poliovirus from the index AFP case or any of the contacts.

### Non-polio AFP

There were no polio-confirmed cases registered in Lebanon between 2009 and 2019, and all non-polio AFP cases were classified according to the final diagnosis. AFP can be caused by several diseases, including Guillain-Barre Syndrome, transverse myelitis, and other pathologies (enteroviruses, peripheral neuropathy, traumatic neuritis).

### Statistical analysis

The yearly incidence rate was obtained by dividing the reported cases of AFP in Lebanon in that year by the total population for the specific age group, including Lebanese and non-Lebanese residents^11^. The reported cases were distributed into three age groups: <5, 5–9, and 10–14 years old. Statistical analyses were performed using the SPSS software version 22.0 (IBM Corp. Released 2013. IBM SPSS Statistics for Windows, Version 22.0. Armonk, NY: IBM Corp.). Tables and figures were generated using the Excel program. A Chi-square test of homogeneity was used to determine whether categories in a variable are equal or not. Incidence rates of AFP in different countries were obtained from a publicly available online database provided by WHO14. The comparison table comprised ten regional countries, including Lebanon, and ten randomly selected countries using a random name picker^13^. A p-value of 0.05 was considered as a cut-off for statistical significance.

This descriptive epidemiological study includes data from public websites (WHO and MOPH's AFP surveillance systems^14^,^15^. As such, an Institutional Review Board (IRB) approval was not needed.

## Results

### Increase in AFP incidence rate until 2015 Incidence

Between 2009 and 2019, the number of cases of AFP in Lebanon reached a total of 631. These cases were not uniformly distributed over the years (p<0.001). The number increased moderately from 8 cases in 2009 to 50 cases in 2014 and abruptly attained a peak of 113 cases in 2015. This number slightly diminished in the following years (Table 1). A compound annual growth rate of 24.23% was recorded in the 11-years period.

**Table 1:** Incidence rate (IR) of AFP per 100,000 children under 15 years old in Lebanon between 2009 and 2019

Year	Incidence	Population (<15)	IR
**2009**	8	1,274,414	0.63
**2010**	19	1,278,703	1.49
**2011**	22	1,347,548	1.63
**2012**	24	1,450,279	1.65
**2013**	33	1,575,438	2.09
**2014**	50	1,696,108	2.95
**2015**	113	1,785,888	6.33
**2016**	111	1,812,365	6.12
**2017**	75	1,813,071	4.14
**2018**	89	1,789,955	4.97
**2019**	87	1,753,120	4.96
**Average**	57.4	1,597,899	3.36

### High incidence of AFP in Mount Lebanon Governorates

Concerning the geographical distribution of AFP, the number of cases throughout these 11 years was evenly scattered among governorates excluding Mount-Lebanon (p=0,250), varying between 49 (Beirut) and 76 (Bekaa) (Table 2). However, a considerable total of 198 cases was recorded in Mount-Lebanon, resulting in a non-homogeneous statistical distribution (p<0.001).

**Table 2:** Incidence (and percentage) of AFP in Lebanon between 2009 and 2019 according to geographic location, age, clinical conditions, follow-up, and final diagnosis

Variable	Year											
	2009	2010	2011	2012	2013	2014	2015	2016	2017	2018	2019	Total
**Governorate**												
Akkar	0	2	2	2	2	1	12	9	7	11	5	**53 (8.4)**
Baalbeck-Hermel	0	2	0	3	2	5	12	9	12	6	9	**60 (9.5)**
Beirut	2	6	2	4	3	2	5	8	8	5	4	**49 (7.8)**
Bekaa	1	0	0	2	4	5	12	10	8	18	16	**76 (12.0)**
Mount-Lebanon	2	5	11	8	12	17	35	45	15	23	25	**198 (31.4)**
Nabatieh	0	2	3	2	1	4	14	12	9	6	10	**63 (10.0)**
North	1	2	2	1	3	11	12	7	4	13	9	**65 (10.3)**
South	2	0	2	2	6	5	11	11	12	7	9	**67 (10.6)**
**Age**												
<5	3	7	6	4	20	19	48	41	33	41	34	**256 (40.6)**
5–9	3	7	7	15	7	18	39	44	27	34	38	**239 (37.9)**
10–14	1	5	9	3	6	13	25	25	15	14	15	**131 (20.8)**
**Clinical Condition**												
Fever	0	6	5	5	8	18	63	71	45	54	65	**340 (53.9)**
Asymmetry of Paralysis	3	8	3	5	3	12	33	31	28	19	23	**168 (26.6)**
Rapid Progress	7	14	13	14	25	32	55	85	55	82	75	**457 (72.4)**
**Follow up**												
Death	1	1	1	0	0	1	5	3	2	1	1	**16 (2.5)**
No Residual Weakness	5	9	10	8	29	24	61	74	56	62	44	**382 (60.5)**
Residual Weakness	2	6	6	8	1	6	23	30	16	24	15	**137 (21.7)**
**Diagnosis**												
Guillain-Barre	6	8	12	16	21	25	31	28	13	22	13	195 (30.9)

### AFP cases highest among children under 5 years

The number of cases varies depending on the age group (p<0.001). Effectively, 239 cases out of 631 were observed in children between 5 and 9 years of age (4,14 per 100,000), and 256 cases were observed in children under five years of age (4.43 per 100,000). These numbers declined to 131 in the 10 to 14-years-old age group (2.17 per 100,000).

### Clinical aspect of AFP in Lebanon

From 2009 until 2014, more patients had no fever at the onset of the disease compared to those who did (Table 2). From 2015 until 2019, when the yearly rates of AFP increased, more patients suffered from fever at the onset of the disease compared to those who did not. In total, the presence of fever at onset was not statistically homogeneous (p=0.003). The p-value is <0.001 if the unspecified cases are taken as a third modality.

In every year observed, the number of patients who did not suffer from asymmetric paralysis exceeded the number of patients who did suffer from it (p<0.001). Nevertheless, the ratio of patients not suffering from asymmetric paralysis over the patients suffering from it was variable throughout the 11 years of the study, reaching a maximum of 10 in 2013 and considerably lower fluctuating values in the remaining years.

Furthermore, a large proportion of patients experienced rapid progress within four days (p<0.001). This observation was most prominent in 2018 when the ratio of patients who progressed rapidly within four days over those who did not attain 11.7 per 100,000. This ratio was the lowest in 2011 when it reached 1.6 per 100,000.

### Follow up

#### Low AFP residual disease

After follow-up, it was noted that 382 out of the total 631 cases (>50%) felt no residual weakness, while 137 cases still felt residual weakness (Table 2). Twelve cases were recorded to be dead.

### Guillain-Barre accounted for a third of AFP cases

The cases of AFP recorded in Lebanon between 2009 and 2019 were diagnosed to be non-related to polio. 32.1% of these non-polio cases were attributed to Guillain-Barre, 1.6% to transverse myelitis, and the majority, which constitutes 62.0%, were attributed to other causes (Table 2). No cases were linked to acute myositis, non-polio myelitis, or vaccine-associated paralytic poliomyelitis (VAPP).

### High number of AFP among patients with 4 doses of vaccine

**Figure 1:**
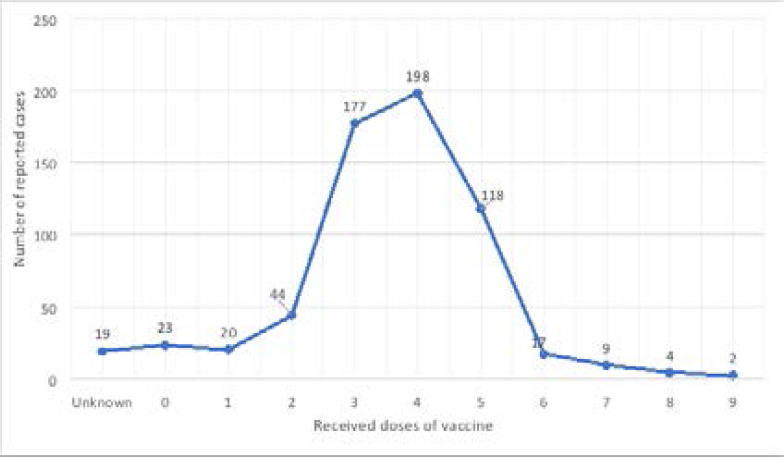
Distribution of AFP cases according to the received doses of IPV/OPV.

The total number of cases recorded from 2009 to 2019 was moderately constant for patients who received 0, 1, 2, 6, 7, 8, and 9 OPV/IPV doses. Forty-four cases were observed at most in the case of 2 vaccination doses, and only 2 cases were noted throughout the 11 years of the study at nine vaccination doses. However, a peak was observed when three to five doses were given, reaching a maximum of 198 total cases at four doses (Figure 1). Statistically, the number of vaccine doses appears to be significant (p<0.001).

### Comparison with other countries: Lebanon's relatively low incidence

According to the MOPH, Lebanon registered 631 cases of AFP between 2009 and 2019, averaging an incidence rate of 3.36 cases per 100,000 individuals under 15 years of age. Compared to selected regional countries (Table 3), that number is lower than the average (5.08). Pakistan registered the highest average rate (11.96), while Saudi Arabia registered the lowest average rate (2.67). Moreover, only Pakistan presented cases of wild poliovirus with 1117 cases throughout the 11 years. Randomly selected countries showed average rates varying between 0.71 (Serbia) and 13.62 (Nigeria) between 2009 and 2019. The European countries, Poland and Serbia, registered the lowest average rate among all selected countries (0.8 and 0.71 respectively). Five of the ten randomly selected countries presented cases of wild poliovirus infections, with the highest number of cases being in India (784), followed by Nigeria (656), and the lowest in Burundi (2). Compared to the randomly selected countries, Lebanon appears to have an under-average (4.52) non-polio AFP rate, albeit six of the selected countries have lower average rates.

**Table 3:** Average incidence rates of AFP and total polio cases in regional and randomly selected countries between 2009 and 2019

	Country	Average AFP Cases	Average non-polio AFP Rate	Total Wild Polio Cases
**Regional** **Countries**	Algeria	318.5	3.17	-
	Bahrain	16.1	8.44	-
	Egypt	1,117.9	4.21	-
	Iran	747.6	3.78	-
	Jordan	69.7	3.12	-
	Kuwait	42.1	5.63	-
	Lebanon	57.4	3.36	-
	Pakistan	7,509.7	11.96	1,117
	Qatar	9.0	4.54	-
	Saudi Arabia	235.7	2.67	-
**Selected** **Countries**	Argentina	181.2	1.77	-
	Bolivia	39.2	1.08	-
	Burundi	114.8	2.40	2
	Congo	152.7	6.35	442
	Cote d'Ivoire	385.7	4.46	62
	India	49,576.7	13.03	784
	New Zealand	8.73	1.03	-
	Nigeria	9,912.36	13.62	656
	Poland	43.70	0.80	-
	Serbia	13.6	0.71	-

## Discussion

### Overall findings

As depicted in the results section, data analysis revealed an overall positive growth rate. The incidence of AFP increased between 2009 and 2015 and was followed by a modest decrease till 2019. Most cases were children under ten years of age. Geographically, the most common governorate for AFP cases was Mount-Lebanon, probably due to its relatively large population and its crowdedness. The third of AFP cases were attributed to Guillain-Barre, while the majority of cases were from other causes. Poliomyelitis incidence was very low. Moreover, two out of fivehildren experienced residual weakness after follow-up. A higher incidence of AFP was observed among patients receiving four vaccination doses, and the curve shows a bell-like distribution. Furthermore, Lebanon seems to have a low average incidence rate compared to other countries.

### Trends of AFP

The incidence rate of AFP increased from 0.63 per 100,000 in 2009 to 4.96 per 100,000 in 2019, reaching a peak of 6.33 per 100,000 in 2015. The remarkable rise in the number of cases described in the results section coincides with the Syrian refugee crisis that led up to 1.5 million refugees from Syria seeking shelter in Lebanon. The population of Lebanon has remarkably increased by 30% because of the influx of Syrian refugees. That influx is highly skewed towards infant and children age groups, unlike the current demographic trends in aging in Lebanon. Studies have shown that Syrian refugees in Lebanon live in poor conditions and suffer mostly from communicable diseases and diseases affecting women^16^. Despite efforts to provide healthcare to these vulnerable populations, outbreaks of many infectious diseases took place^17^, and cases of AFP kept increasing throughout the years. The incidence rate stabilized from 2017 till 2019 ranging from 4.14 and 4.96 per 100,000, respectively. In this period of time, the refugee influx has reached a plateau, which explains the decline of incidence rates from 6.33 per 100,000 in 2015 to 4.96 per 100,000 in 2019.

### Future projections

As for future projections, it should come as no surprise that the incidence would get back to the pre-2011 situation, as the refugee population is stabilizing and blending into the Lebanese context, thus, becoming vulnerable to the microbiological realities just the same way as the Lebanese population. Prevention of outbreaks and implementation of vaccination programs throughout the country are needed, especially with the progressively deteriorating economy. In addition, the increase in cases can be partly explained by the enhanced efficiency of the surveillance programs throughout their decade of experience. Lastly, further screening of the vaccination program could reveal possible causes of vaccine-induced AFP due to defective vaccines.

### Poliomyelitis Surveillance System

AFP Surveillance systems report each year the number of cases of polio and non-polio AFP and are used as a proxy for measuring the validity of the polio surveillance system^14^,^18^. To evaluate the sensitivity of the surveillance systems, the WHO set a threshold of at least 1 case per 100,000 individuals under 15 years of age that surveillance systems must register for non-polio AFP cases^19^. Besides 2009, rates of more than 1/100,000 were registered every other year, indicating an acceptable performance of the MOPH surveillance system according to the standard set by the WHO. In recent years, especially between 2015 and 2019, the incidence of non-polio AFP reached new heights, with the incidence rate increasing by more than 100% between 2014 and 2015 and maintaining a relatively high number in the following years. These findings suggest an adequate sensitivity of the national surveillance system. The AFP cases are expected to increase in the upcoming years to reach around 235 cases in 2030.

### AFP distribution by age groups

The study reported that throughout the 11 years, there were almost as many cases of non-polio AFP in children under five years of age (40.6%) as there was in children between five and nine years old (37.9%), while the 10–14 years' group represented a minority in the study (20.8%) (Table 2). The remaining cases were categorized as unspecified (0.7%). These numbers showed to be similar to the results recorded in a study conducted in Italy between 1997 and 2007^20^. However, similar studies in Jordan, Iran, Congo, and Bangladesh^21^–^24^ suggested that the incidence was significantly higher in children under five years old.

### Causes of AFP in Lebanon

With the advances in the eradication of poliomyelitis, GBS has become the most common cause of AFP in the world^20^. In this study, 195 out of 608 AFP cases were diagnosed as GBS (32.1%) (Table 2), and 377 out of the 608 cases (62.0%) were classified as other diagnoses, which included enteroviruses, peripheral neuropathies, traumatic neuritis, and many more. Compared to similar studies, the percentage of GBS diagnoses is relatively low. For instance, studies in Iran, Bangladesh, Australia, and Iraq showed that the frequency of GBS as the underlying cause of AFP is more than 50%^22^, ^24^–^26^.

### Vaccination rate

This study revealed that 83.2% of AFP cases received at least 3 doses of OPV/IPV vaccine, 13.8% received less than three doses (out of which 26.4% were not vaccinated at all), and the remaining cases were unspecified (3.0%). This vaccination rate of 83.2% is considered significantly high compared to the same rate in Congo (58.9%) between 2008 and 2014^21^, but relatively low compared to the 98.3% found in Ikwa Ibom state, Nigeria^7^.

### Correlation of vaccination with AFP rate

Moreover, the study showed that most AFP patients were individuals having received three, four, and five vaccine doses. The incidence according to vaccine dosage follows a bell-shaped curve almost every year, where most cases have received between three and five doses of IPV or OPV (Figure 1). Interestingly, GBS is known to be caused by vaccination/inoculation and other infectious/immunological causes. Naeem et al. (2016) reported a Guillain Barre Syndrome (GBS) after oral polio vaccination^28^. Hence, these vaccination doses (between three and five vaccines) could cause vaccine-associated AFP. Future studies should be conducted to assess the validity and strength of the correlation between the number of AFP cases and the vaccination doses and possibly obtain an explanation for this finding.

### Comparison with other countries

According to the MOPH, Lebanon registered 631 cases of AFP between 2009 and 2019, averaging an incidence rate of 3.36 cases per 100,000 individuals under 15 years of age. Compared to selected regional countries (Table 3), that number is lower than the average (5.08). Pakistan registered the highest average rate (11.96), while Saudi Arabia registered the lowest average rate (2.67). Moreover, only Pakistan presented cases of wild poliovirus with 1117 cases throughout the 11 years. Randomly selected countries showed average rates varying between 0.71 (Serbia) and 13.62 (Nigeria) between 2009 and 2019. The European countries, Poland and Serbia, registered the lowest average rate among all selected countries (0.8 and 0.71 respectively). Five of the ten randomly selected countries presented cases of wild poliovirus infections, with the highest number of cases being in India (784) followed by Nigeria (656), and the lowest in Burundi (2). Compared to the randomly selected countries, Lebanon appears to have an under-average (4.52) non-polio AFP rate, despite the fact that six of the selected countries have lower average rates.

### Study limitations

We focused in this study on AFP cases occurring in children from 0 to 15 years. This study presented a lack of specification in the data, such as the absence of specification of the neurologic exam of the different presentations of AFP (limb involved by the paralysis, etc.) and a lack of detailed description of the neurologic examination in the patients who improved spontaneously and those who did not, or worsened. Another shortfall in the study was the lack of a gender specification for the cases, which would have allowed a more significant comparison with previous similar studies. Under-reporting of AFP cases would be unusual in this study due to the sizeable involvement of international organizations with the refugee population and their focus on preventable infectious diseases such as Polio and Measles. Finally, due to the unexpected demographic dynamics and political situation of Lebanon, it would have been inaccurate to provide statistical projections of incidence rates in future years.

## Conclusion

The data provided by the MOPH was evaluated in terms of incidence rates of AFP in Lebanon, age distribution, causes of the disease, and vaccination doses, in order to get a better understanding of the disease and improve the plans of action towards its eradication. AFP incidence rate increased during the Syrian refugee crisis and is expected to stabilize in the upcoming years; still, Lebanon holds a lower-than-average incidence rate compared to regional and random countries. In the light of the ongoing refugee crisis and the deteriorating socio-economical norms, physician/patient awareness, prevention, and vaccination are crucial in flattening the curve of AFP and controlling the disease.

## List of Abbreviations

AFP: Acute Flaccid Paralysis
ESU: Epidemiological Surveillance Unit
GBS: Guillain Barre Syndrome
MOPH: Ministry of Public Health
IPV: Inactivated polio vaccine
OPV: Oral polio vaccine
WHO: World Health Organization

## Declarations

### Availability of data and material

This descriptive epidemiological study includes data from public websites (WHO and MOPH's AFP surveillance systems).
